# Supplementary material for: Revealing the aging process of solid electrolyte interphase on SiOx anode
Source: Nat Commun. 2023 Sep 28;14:6048. doi: 10.1038/s41467-023-41867-6 (PMC10539371; doi:10.1038/s41467-023-41867-6)
Supplement: Supplementary file 1 — Supplementary information [file 41467_2023_41867_MOESM1_ESM.pdf]

Supplementary information

Revealing the Aging Process of Solid Electrolyte Interphase on SiO_x Anode

Guoyu Qian[1,2,11], Yiwei Li[1,11], Haibiao Chen[1,3], Lin Xie[4], Tongchao Liu[5], Ni Yang[1], Yongli Song[1], Cong Lin[1,6], Junfang Cheng[7,8], Naotoshi Nakashima[7], Meng Zhang[9], Zikun Li[9], Wenguang Zhao[1], Xiangjie Yang[2], Hai Lin[1], Xia Lu[2], Luyi Yang,[1]*, Hong Li[10], Khalil Amine[5], Liquan Chen[10] and Feng Pan[1]*

[1]School of Advanced Materials, Peking University, Shenzhen Graduate School, Shenzhen, China. [2]School of Materials, Sun Yat-sen University, Shenzhen, China. [3]Institute of Marine Biomedicine, Shenzhen Polytechnic, Shenzhen, China. [4]Department of Physics, Southern University of Science and Technology, Shenzhen, China. [5]Chemical Sciences and Engineering Division, Argonne National Laboratory, Argonne, IL, USA. [6]Department of Applied Biology and Chemical Technology, The Hong Kong Polytechnic University, Hong Kong S.A.R. [7]International Institute for Carbon-Neutral Energy Research (WPI-I2CNER), Kyushu University, Fukuoka, Japan. [8]SJTU Paris Elite Institute of Technology, Shanghai Jiao Tong University, Shanghai, 200240 P. R. China. [9]BTR New Material Group Co., Ltd., Shenzhen, China. [10]Institute of Physics, Chinese Academy of Sciences, Beijing, China. [11]These authors contributed equally.

*E-mail: yangly@pkusz.edu.cn; panfeng@pkusz.edu.cn.

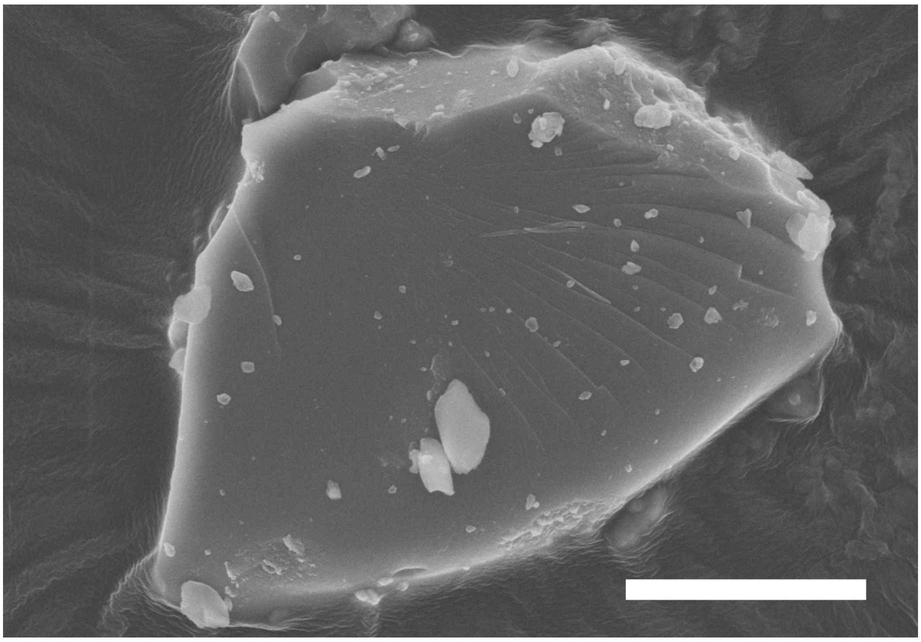

Supplementary Fig. 1 Morphology of SiO_x particle. SEM image of a pristine SiO_x particle, the scale bar = 2 μm.

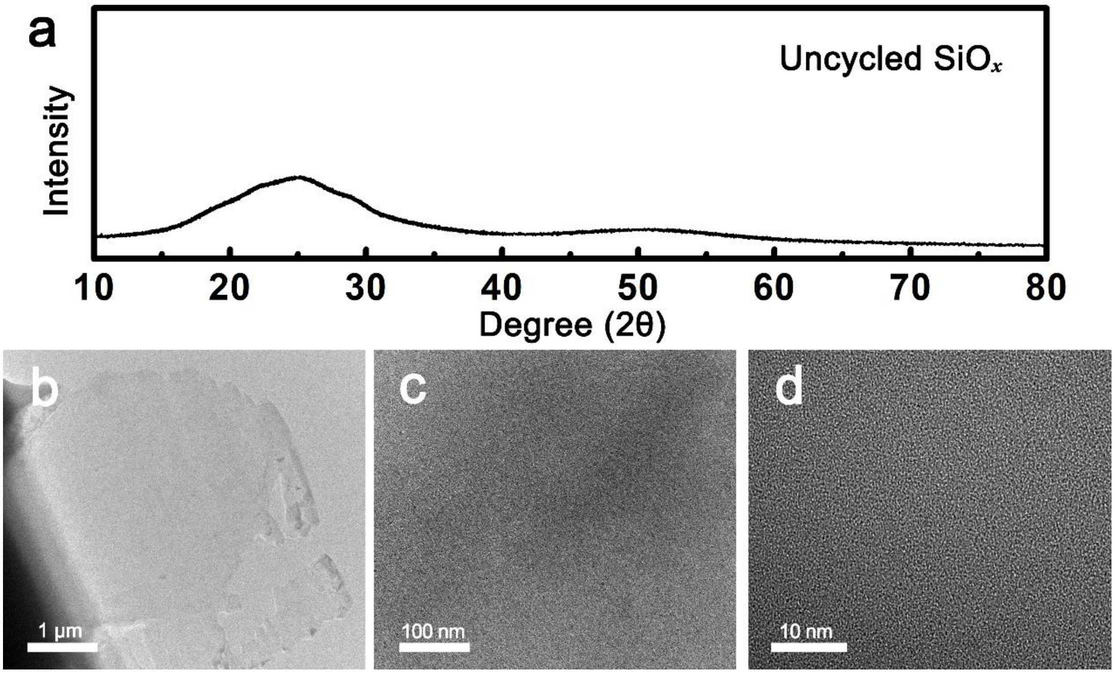

Supplementary Fig. 2 Basic characterization of SiO_x material. **a** XRD pattern of pristine SiO_x. **b-d** TEM images of the a pristine SiO_x particle in different magnification, which shows no crystalline phase existed in the samples.

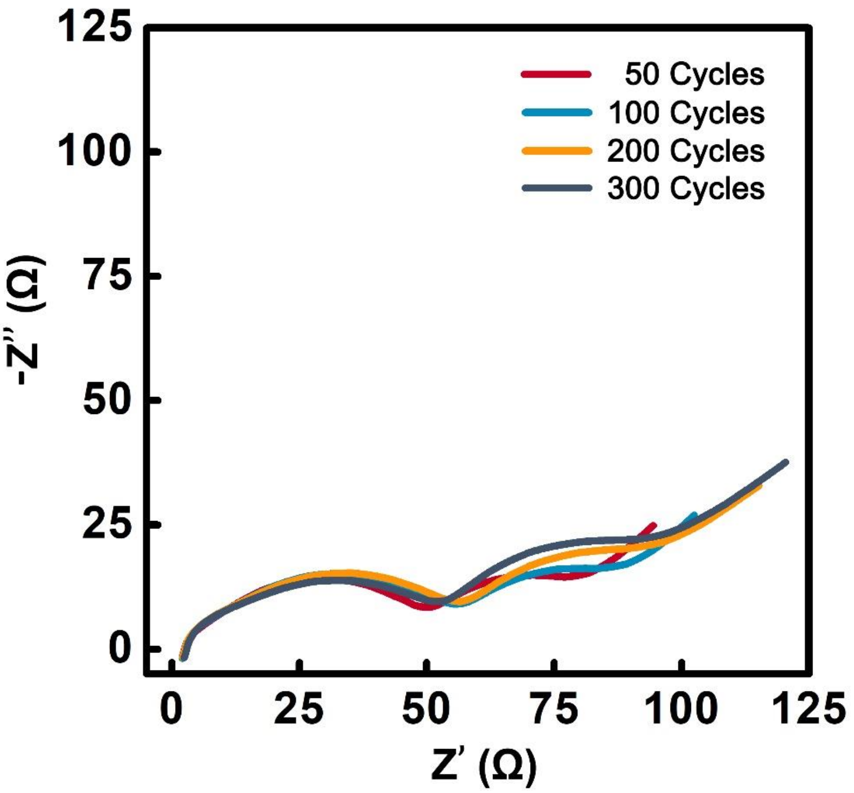

Supplementary Fig. 3 Electrochemical impedance results of SiO_x electrode. Nyquist plots of a SiO_x||Li half-cell after 50, 100, 200, 300 cycles.

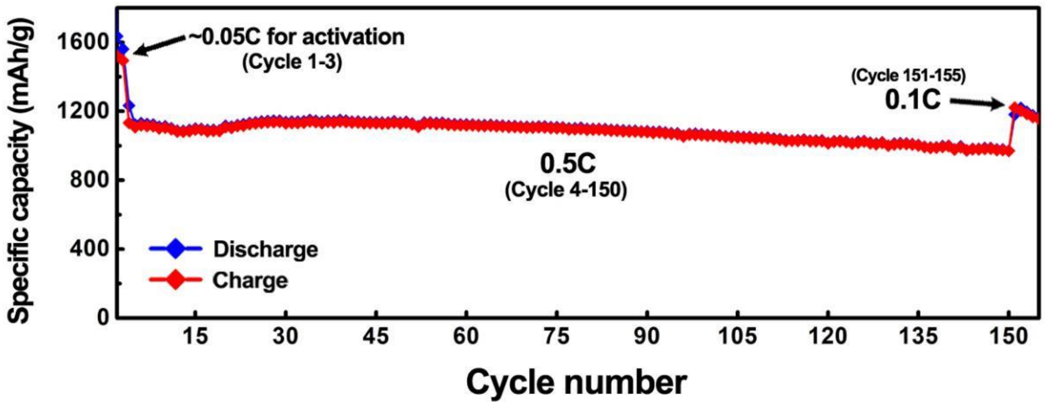

Supplementary Fig. 4 Charge/discharge cycling test of the SiO_x||Li half-cell in different current density. The battery was first activated at a low current density 75 mA g^{-1} for 3 cycles. Then the battery was cycled at 750 mA g^{-1} until the 150th cycle. Afterwards, by applying a lower current of 150 mA g^{-1} to the half-cell after long-term cycling (150 cycles) for 5 cycles, a specific capacity of 1155 mAh g^{-1} could be restored.

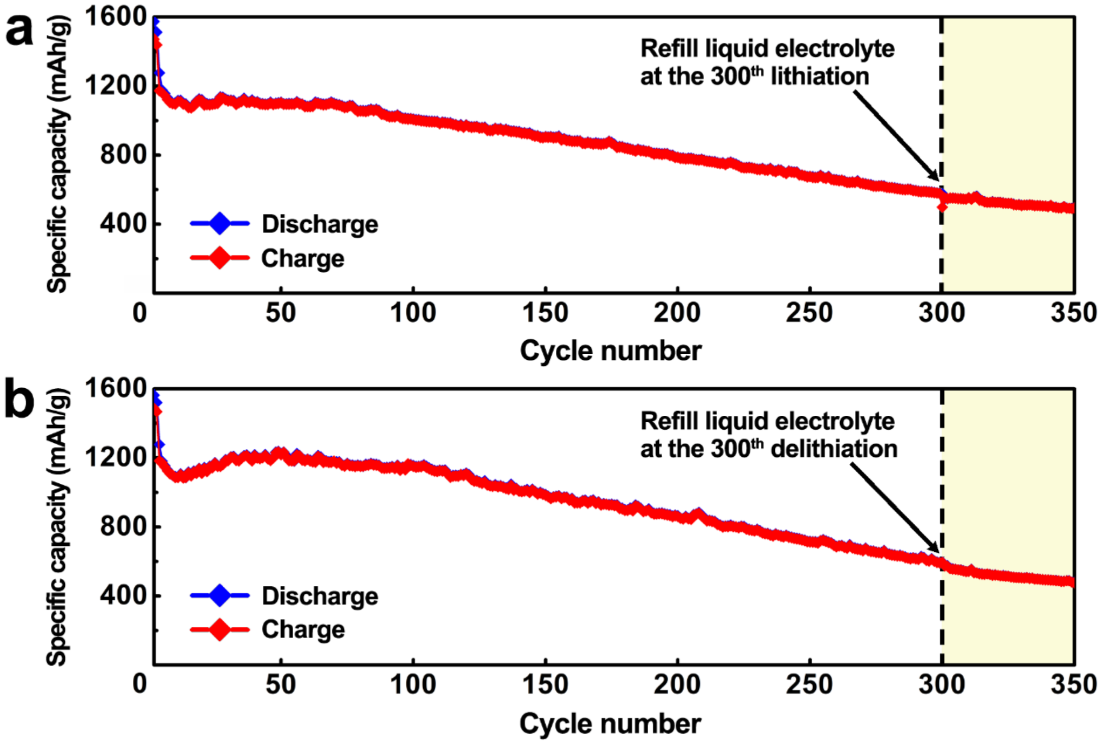

Supplementary Fig. 5 Cycling performance of SiO_x||Li half-cells at 750 mA g^{-1}. The half-cell was disassembled, refilled with electrolyte and cycled again after **a** the 300th lithiation and **b** the 300th delithiation.

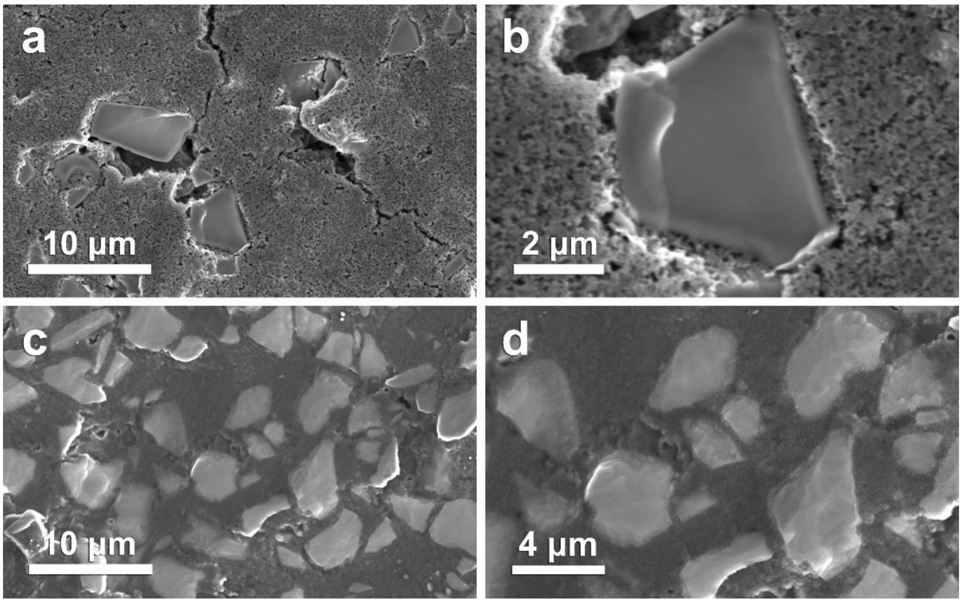

Supplementary Fig. 6 SEM images of SiO_x electrode under different states. The cross-sectional SEM images of the SiO_x electrode (**a-b**) before cycling, and (**c-d**) after 1st delithiation in different magnification.

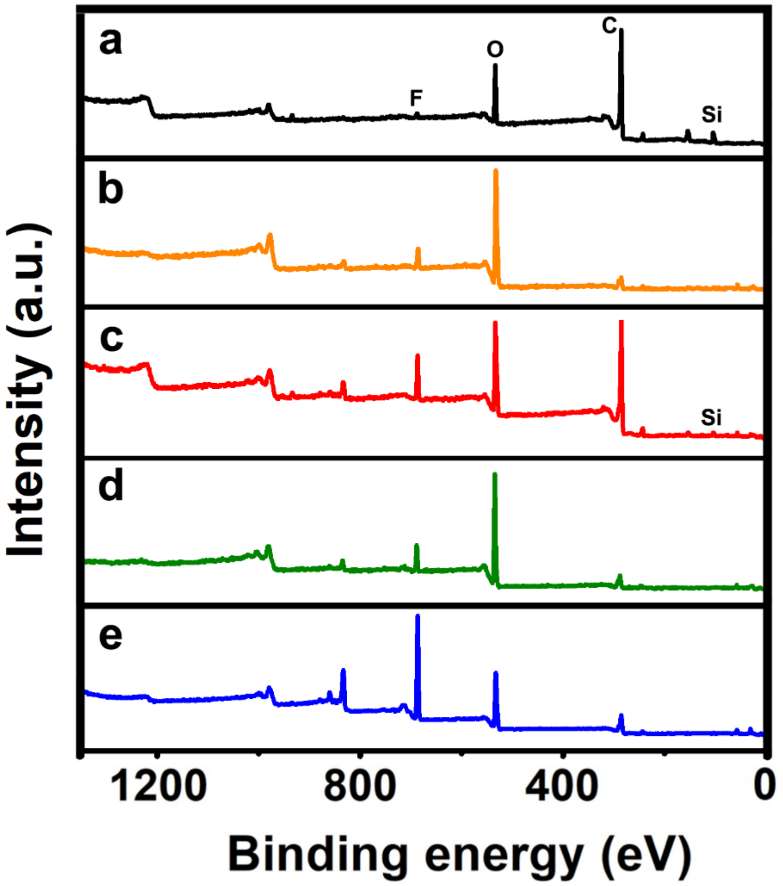

Supplementary Fig. 7 Full XPS spectra of the SiO_x electrode. a before cycling; **b** after 1st lithiation; **c** 1st delithiation; **d** 300th lithiation and **e** 300th delithiation.

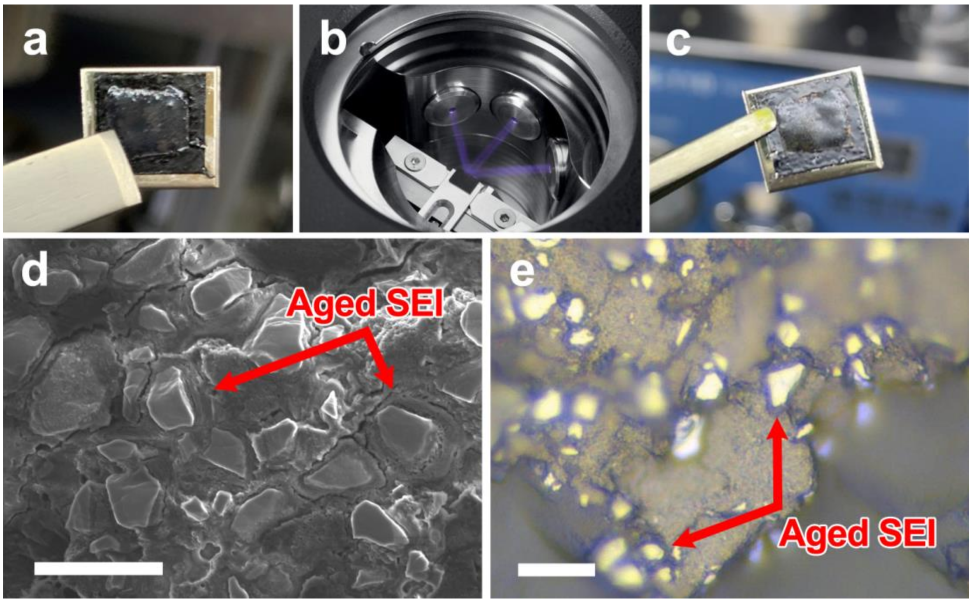

Supplementary Fig. 8 Sample preparation and images of SiO_x electrode. a The electrode surface was embedded in the resin. **b** The specimen was gradually polishing with a triple Ar ion-beam milling system. **c** The polished electrode showed a super-flat top surface. **d-e** The cross-sectional images of the topmost particles in the electrode at the 300th delithiated state, investigated by SEM and optical microscope, respectively. The scale bar = 10 μm.

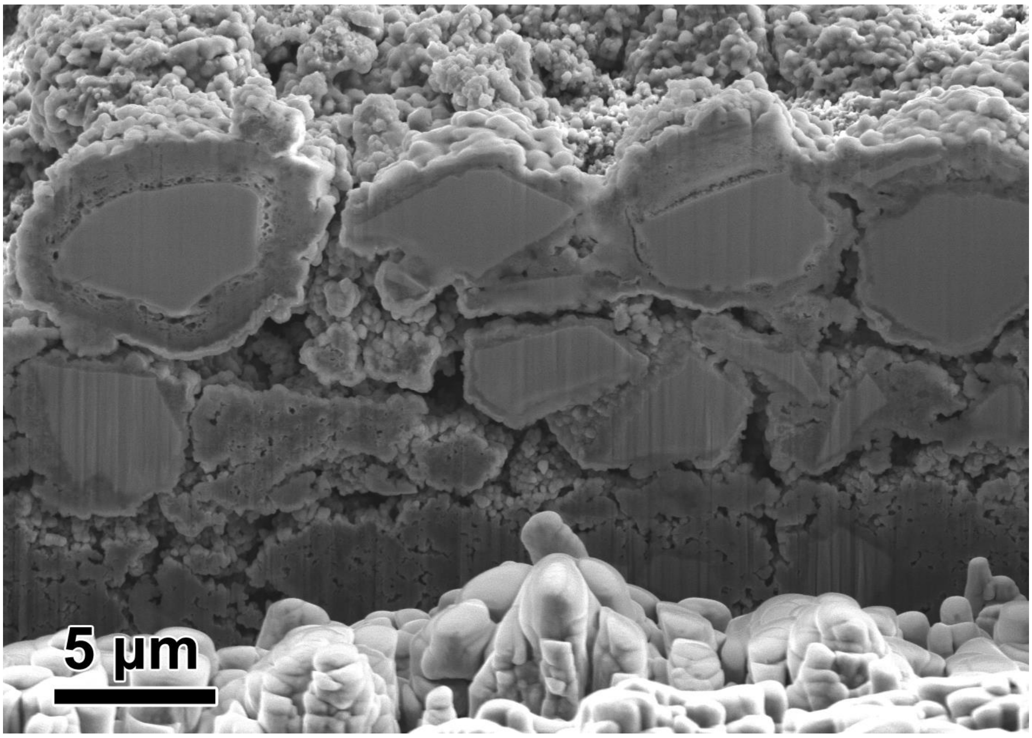

Supplementary Fig. 9 Morphology of a cycled SiO_x (x=0.98) electrode. Cross-sectional SEM image of a SiO_x (x=0.98) electrode after 300 cycles.

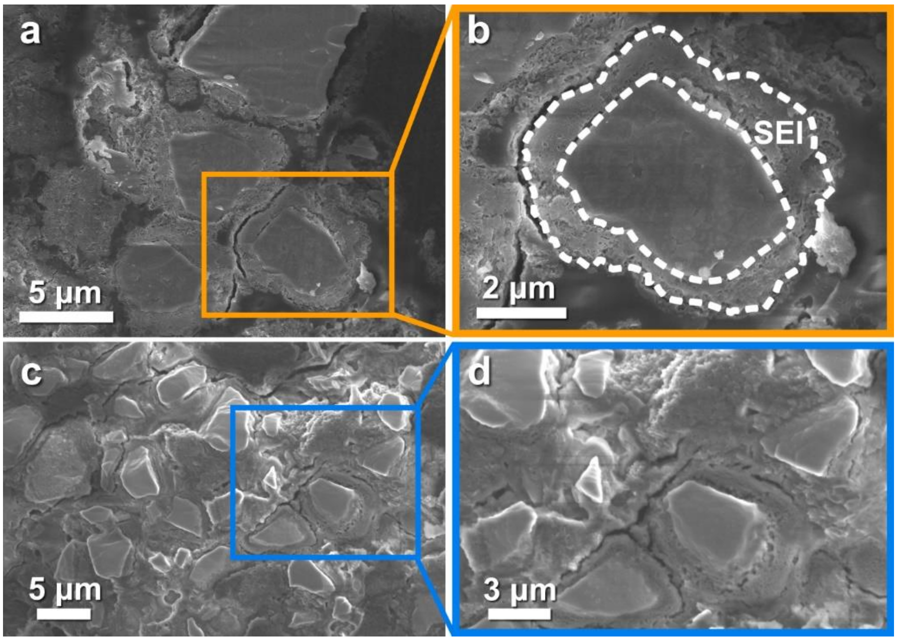

Supplementary Fig. 10 Morphology information of SEI prepared through different methods. Cross-sectional SEM images of SiO_x anode after 300th delithiation prepared from **a** cryo-ultramicrotomy and **c** FIB. The corresponding enlarged figures are displayed in **b** and **d** respectively.

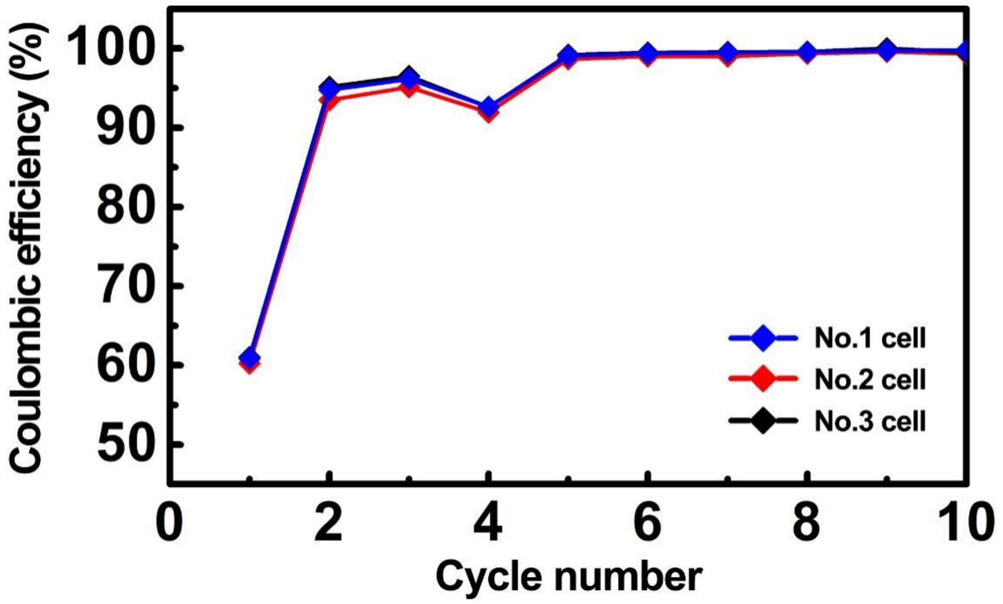

Supplementary Fig. 11 Coulombic efficiency fluctuations of SiO_x electrodes. Variation of 3 different SiO_x||Li half-cells at 750 mA g^{-1} during the intial 10 cycles.

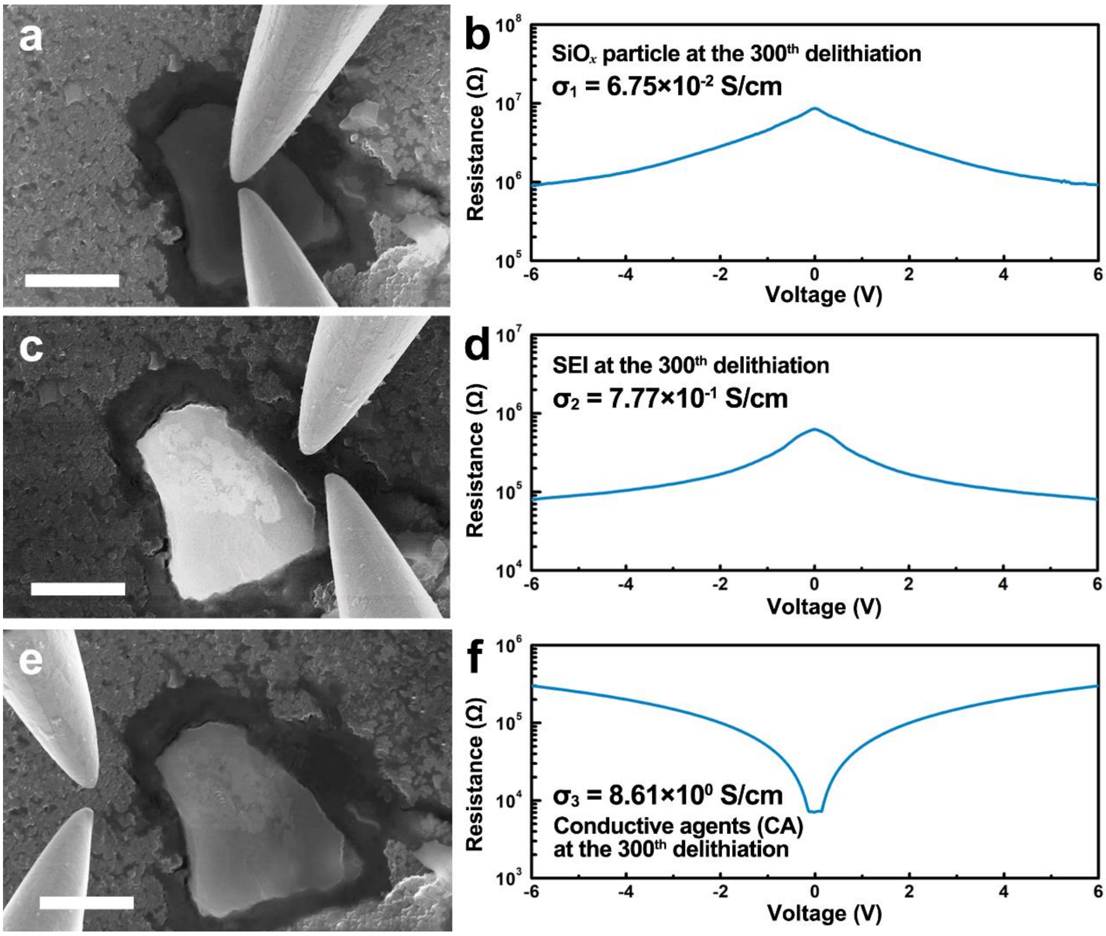

Supplementary Fig. 12 Conductivity measurement of different regions in a SiO_x electrode. Measuring the electronic conductivity of different areas on a section of long-cycled SiO_x electrode: **a,** the SiO_x particle; **c,** the SEI region; **e,** the carbon black-binder mixture. Resistivity vs. voltage curves and their conductivities in **b,** the SiO_x particle; **d,** the aged SEI; **f,** CA-binder regions. The scale bar = 2 μm.

Supplementary Note 1 The calculation process of electronic conductivity.

The resistance changes dV/dI can be used to determine the sample conductivity σ by using Laplace's equation[1,2].

$$\frac{dV}{dI} = \frac{\rho}{\pi} \times \left(\frac{1}{r} - \frac{1}{3d-r}\right)$$

$$\sigma = \left(\frac{dV}{dI}\right)^{-1} \times \frac{1}{\pi} \times \left(\frac{1}{r} - \frac{1}{3d-r}\right)$$

where ρ is the sample resistivity, d is probe spacing and r is tip contact radii.

For the SiO_x particle and the aged SEI specimen, the $\frac{dV}{dI}$ are obtained from the curve at a high bias (at ±6 V) where the contact Schottky Barriers between the probes and the material are overcome; For the carbon black-binder (conductive agents) specimen, the $\frac{dV}{dI}$ is obtained from the curve near the zero bias (at ±0.1 V) where the concentration of charge carrier has not reached saturation.

a. SiO_x particle

$\frac{dV}{dI} = R_{6V} = 8.98 \times 10^5\ \Omega$, $d_1 = 361$ nm, $r_1 = 50$ nm

$\sigma_1 = \left(\frac{dV}{dI}\right)^{-1} \times \frac{1}{\pi} \times \left(\frac{1}{r} - \frac{1}{3d-r}\right) = 6.75 \times 10^{-2}$ S/cm

b. Aged SEI

$\frac{dV}{dI} = R_{6V} = 7.97 \times 10^4\ \Omega$, $d_2 = 626$ nm, $r_2 = 50$ nm

$\sigma_2 = \left(\frac{dV}{dI}\right)^{-1} \times \frac{1}{\pi} \times \left(\frac{1}{r} - \frac{1}{3d-r}\right) = 7.77 \times 10^{-1}$ S/cm

c. Conductive agents

$\frac{dV}{dI} = R_{0.1V} = 7.20 \times 10^3\ \Omega$, $d_3 = 582$ nm, $r_3 = 50$ nm

$\sigma_3 = \left(\frac{dV}{dI}\right)^{-1} \times \frac{1}{\pi} \times \left(\frac{1}{r} - \frac{1}{3d-r}\right) = 8.61 \times 10^0$ S/cm

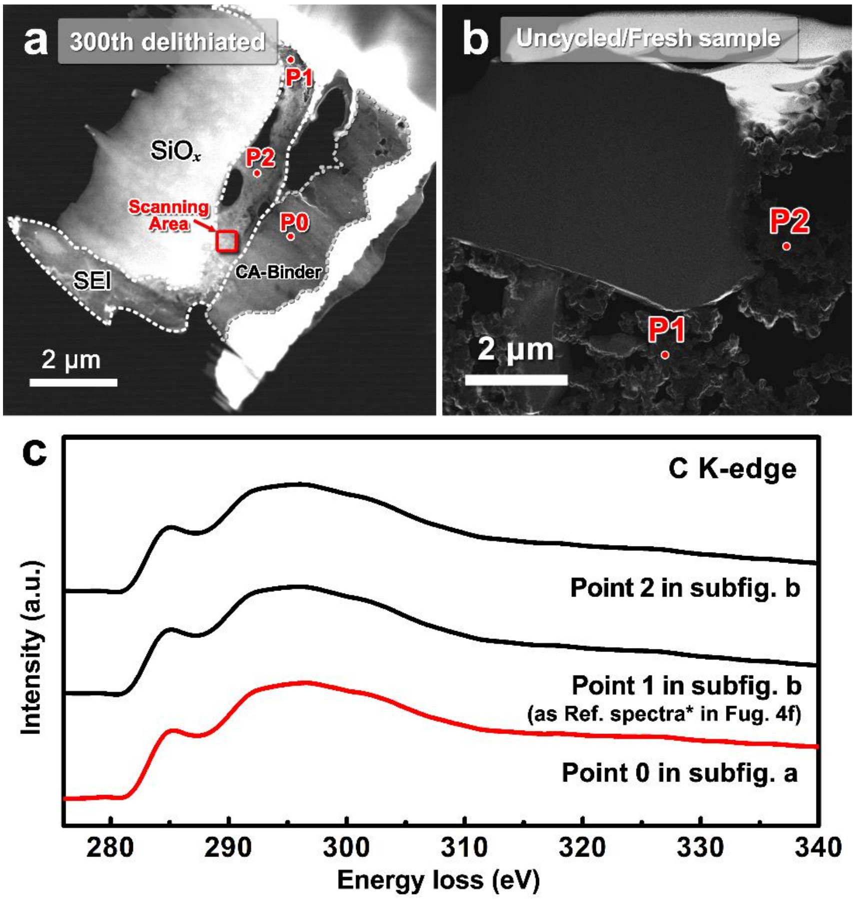

Supplementary Figure 13 Electron transparent FIB lift-out lamellae. a Cycled and **b,** pristine SiO_x particle and its SEI with surrounding CA-binder domain. **c,** EELS spectra of the C K-edge from P1, P2 in the uncycled CA-binder domain and P0 in the CA-binder domain of cycled SiO_x.

To determine the distribution of CA-binder, we normalized the curve based on the peak between the region of 380-420 eV, which is away from the near-edge peaks of carbon. Next, we used Point 1 (CA-binder domain) in Supplementary Fig. 13b as the reference (x_i) to evaluate the resemblance between a certain point (y_i) with the reference at the region of near-edge fine structure of carbon (280-295 eV) based on mean square error method shown as below:

$$M = \frac{\sum_{i=1}^{n}(y_i - x_i)^2}{N}$$

A smaller M value represents a higher resemblance between this point, hence the chemical composition of this point in SEI is closer to the CA-binder domain.

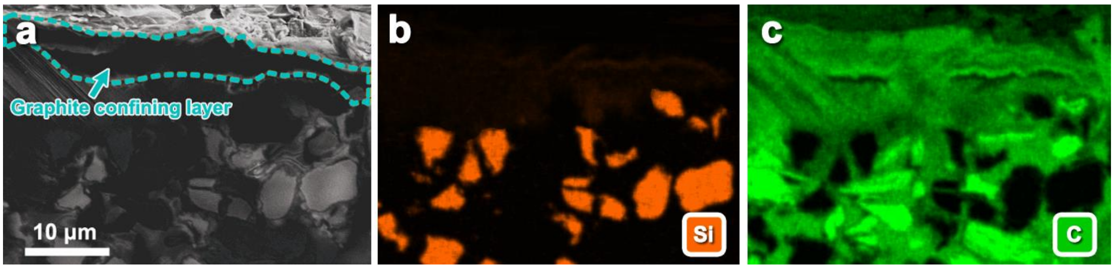

Supplementary Figure 14 Morphology and elemental map of graphite coated SiO_x anode. a, Cross-sectional SEI image of a graphite coated anode after 300 cycles and the corresponding EDS mapping of **b,** Si and **c,** C. It can be seen that a layer (~5 um) with only carbon is firmly covered on the electrode, indicating the graphite coating layer remain on the top after cycling.

Supplementary references

1. Polly, C. M. et al. Microscopic four-point-probe resistivity measurements of shallow, high density doping layers in silicon. *Appl. Phys. Lett.* 101, 262105 (2012).

2. Schroder, D. K. Semiconductor Material and Device Characterization (John Wiley & Sons, 1990), p. 2.
