# Supplementary material for: Revealing the aging process of solid electrolyte interphase on SiOx anode
Source: Nat Commun. 2023 Sep 28;14:6048. doi: 10.1038/s41467-023-41867-6 (PMC10539371; doi:10.1038/s41467-023-41867-6)
Supplement: Supplementary file 3 — Description of additional supplementary files [file 41467_2023_41867_MOESM3_ESM.pdf]

<u>Description of Additional Supplementary Files</u>

Supplementary Movie 1: Movies of SiOx particles under different cycle states being gradually sliced by FIB.
